# Acute Skin Wounds Treated with Mesenchymal Stem Cells and Biopolymer Compositions Alone and in Combination: Evaluation of Agent Efficacy and Analysis of Healing Mechanisms

**DOI:** 10.3390/pharmaceutics13101534

**Published:** 2021-09-22

**Authors:** Ekaterina Silina, Victor Stupin, Natalia Manturova, Vitaly Vasin, Konstantin Koreyba, Petr Litvitskiy, Alexander Saltykov, Zalim Balkizov

**Affiliations:** 1 Institute of Biodesign and Modeling of Complex Systems, I.M. Sechenov First Moscow State Medical University (Sechenov University), 119991 Moscow, Russia; lisovikp@mail.ru (P.L.); absaltykov@yandex.ru (A.S.); 2 Department of Hospital Surgery №1, N.I. Pirogov Russian National Research Medical University (RNRMU), 117997 Moscow, Russia; stvictor@bk.ru (V.S.); vitalikv2010@mail.ru (V.V.); korejba_k@mail.ru (K.K.); balkiz@mail.ru (Z.B.); 3 Department of Plastic and Reconstructive Surgery, Cosmetology and Cell Technologies, N.I. Pirogov Russian National Research Medical University (RNRMU), 117997 Moscow, Russia; manturovanatali@yandex.ru

**Keywords:** biomaterials, biopolymers, nanoparticles, mesenchymal stem cells, experimental dermatology, skin wound, regenerations, wound healing

## Abstract

We studied the efficacy of using mesenchymal stem cells (MSC) and a polymeric compound (based on chitosan and cellulose with integrated cerium dioxide nanoparticles (PCCD)) in wound healing, and to compare the effects with various invasive and external drugs used for the same purpose. Two wounds were made on the backs of each of 112 Wistar rats, removing the skin. Eight groups were studied: Control_0—intact wounds; Control_ss—0.9% NaCl injections; MSC injections; Control_msc—intact wounds on the opposite side of the body from the MSC group; external application of the PCCD; external application of a combination of the drugs PCCD + MSC; DCh –ointment Dioxomethyltetrahydropyrimidine + Chloramphenicol; and DHCB—injections of a deproteinized hemoderivative of calf blood. After 14 days, we evaluated the state and size of the wounds, studied the level of microcirculation, performed a histological study, and identified and counted the different types of cells. The most effective remedy was combination PCCD + MSC. The treatments in the PCCD and MSC groups were more effective than in the DHCB and DCh groups. Invasive drugs and DCh slowed the regeneration process. DHCB did not affect the rate of healing for acute wounds without ischemia during the first week. The proven efficacy of developed polymeric compounds demonstrates the feasibility of further studies in clinical practice.

## 1. Introduction

Questions concerning how a variety of wounds heal and effective medical support of the healing process remain open to debate and of immediate concern. One compelling explanation is the increased number of surgical operations in the world, as well as the increased number of wounds caused by domestic disputes and military actions, which are responsible for a somatically healthy person receiving an acute wound [1,2,3]. Added to this is the trend of increased life expectancy, and hence comorbidity, which is accompanied by a growing prevalence of chronic trophic ulcers of various etiologies that are becoming an acute clinical problem [4,5,6]. Despite the constantly increasing number of drugs and methods used to treat wounds, the problem of effective regeneration has not yet been resolved [5,6,7,8,9,10,11,12]. The results of meta-analyses and systematic reviews of the effectiveness of different agents and methods used to treat wounds demonstrate the lack of a uniform and effective approach, as well as a limited choice of drugs for use in wound treatment [13,14,15,16].

It is obvious that the development of safe and effective new medicines for the treatment of wounds is urgent. Recently, promising results have been demonstrated using multicomponent dressings based on biomaterials such as stem cells [17,18,19,20] and polymers [21,22,23,24,25,26,27,28]. Good preliminary results were shown by studies with the inclusion of chitosan and cellulose in dressings. Cellulose resorption does not occur in the cells because animal cells do not produce cellulose enzymes. The efficacy of cellulose is associated with the maintenance and release of growth factors at the site of injury, which promote the migration and proliferation of fibroblasts and inhibit bacterial growth in the wound [25,26,27,28]. The biopolymer chitosan is known for its heterogeneous functionality, non-toxicity, inertness, non-antigenicity, bioadhesiveness, biocompatibility, biodegradability, and its hemostatic and antimicrobial properties [21,22,23,24]. Metal molecules integrated into biomaterials have demonstrated useful regenerative properties [22,23,24,29]. However, not enough research has yet been done investigating this new direction to provide data that meet all the requirements of evidence-based medicine. In our previous research, we developed new dressing biomaterials for the treatment of wounds containing polymers, mesenchymal stem cells, and nanoparticles of cerium dioxide (CeO_2_), and demonstrated their preliminary safety and efficacy [30,31,32,33,34]. We have chosen CeO_2_ nanoparticles as the metal included in our biomaterials because previous studies have shown that it has low toxicity and high biocompatibility, as well as the ability, depending on the changing pH of the wound, to provide electron transfer through the transition from Ce^3+^ to Ce^4+^ valence and vice versa [35,36,37,38]. This allows the wound environment to become stable by neutralizing reactive oxygen species and other free radicals, providing antioxidant, antibacterial, and anti-inflammatory effects.

This aim of this research was to study the efficacy of using a biopolymeric agent based on chitosan and cellulose with integrated cerium dioxide nanoparticles, mesenchymal stem cells, and these two treatments in combination and to compare the treatment results with control wounds and the efficacy of two registered regenerative drugs (invasive and non-invasive) used for wound healing.

## 2. Materials and Methods

An experimental, preclinical, randomized control comparative study was performed on 112 white male Wistar rats of mature post-reproductive age (9 months old; weight on the wound modeling day 424 ± 55 g).

The wounds were created under general anesthesia (chloral hydrate 300 mg/kg intraperitoneally). On the shaved skin of the back, at the same distance from both sides of the spine, using a special device (RF Patent No. 79701/10.01.2009), square wounds of the same size (11 mm long on all sides) were formed, removing all the skin layers down to the muscle fascia. The average size of the wound areas was 132 ± 13 mm^2^ (Me = 128.8 mm^2^). A total of 224 wounds were modeled.

The data comprise the treatment results of four groups of animals, each of which included two drug treatment groups. Eight pharmacological groups of wounds were studied: (1) Control_0 (C0)—intact wounds healing under a scab without the addition of drugs; (2) Control_ss (Css)—isotonic saline solution (0.9% NaCl) was injected into the wound edges on the day of modeling (Day 0); (3) mesenchymal stem cells (MSC)—a suspension of a progenitor MSC culture obtained from the human umbilical cord at a concentration of 5 × 10^5^ (a total of 100,000 MSC per wound) was injected into the edges of the wounds on the Day 0; (4) Control_msc—intact contralateral wounds on the backs of rats in the MSC group, on the opposite side of the body, without direct drug action (systemic action of MSC); (5) (PCCD)—polymeric compound with cerium dioxide (PCCD); a biological drug containing biocompatible cerium dioxide nanoparticles integrated into a hydrogel matrix, including natural and synthetic polymers of structure-forming polysaccharides, was applied to the surface of the wounds on Days 0, 1, 3, and 7 (To prepare the hydrogel base of the PCCD drug, we added a dry sample of carboxymethylcellulose (20 g), chitosan (2 g; Sigma Aldrich), pectin (2 g; Sigma Aldrich), agar-agar (2 g; Roeper), and sodium alginate (1 g; Sigma Aldrich) to 975 mL of H_2_O. Next, a colloidal solution with a cerium oxide concentration of 0.004 g/L was added dropwise to 25 mL of the prepared gel matrix with constant stirring); (6) PCCD + MSC—a biological product containing PCCD and MSC was applied externally (50,000 cells per wound) on Days 0, 1, 3, and 7; (7) DCh—the combined antimicrobial ointment Levomekol^®^, containing dioxomethyltetrahydropyrimidine (40 mg) and Chloramphenicol (7.5 mg) in 1 g, was applied externally to the wounds on Days 0, 1, 3, and 7; and (8) DHCB—deproteinized hemoderivative of calf blood (Actovegin^®^; 40 mg/mL) was injected into the wound edges on Day 0.

Thus, among theeight8 groups, three received drug injections into the wound edges (in the Css, MSC, and DHCB groups, 0.2 mL of each drug solution was injected via a single puncture on Day 0), three received external application of the drug on the wound surface (in the PCCD, PCCD + MSC, and DCh groups, the agent was applied in a volume of 1 mL per cm^2^ on Days 0, 1, 3, and 7), and the wounds of the rats in the remaining two groups were intact, healing under scabs (C0 and Control_msc). The application technique and the drug volume within the treated wound groups were of the same type and were performed under standard conditions (room temperature 21–23 °C; air humidity 50% ± 5%). All animals shared the same living conditions and standard of care. During the entire study period, the animals were kept in individual cages under standard vivarium conditions with a 12/12 light/dark regime and free access to food and water.

The composition and technology of the drugs PCCD, MSC, and PCCD + MSC have been published previously [30,31,32,33,34].

There were six control research points: Day 0 (wound modeling, measurements, and treatment) and Days 1, 3, 5, 7, and 14 (assessment of the wound healing dynamics). On Days 3, 7, and 14 of the experiment, seven animals from each group were taken out of the experiment under general anesthesia (chloral hydrate 300 mg/kg intraperitoneally).

The study was carried out using visual, instrumental, and laboratory control of modern methods used to assess the effect of drugs following the Guidelines for Preclinical Study of New Pharmacological Substances developed by the Federal State Institution Scientific Center for Expertise of Medical Products and the Federal Service for Healthcare Surveillance and Social Development. The study was approved at the meeting of the Regional Ethics Committee of the Kursk State Medical University under the Ministry of Health of the Russian Federation (Protocol No. 5 dated 2 November 17) and at the Commission of the Pirogov Russian National Research Medical University on control over the maintenance and use of laboratory animals (Protocol No. 5 dated 11 May 2021).

On Days 0, 1, 3, 5, 7, and 14 of the experiment, the animals were examined, weighed (Supra BSS-4095 scales; ±1 g), and the dynamics of the wound size and condition were assessed by measuring the wound area (in mm^2^) using JMicroVision 1.2.7 software (Switzerland) based on photographs of the wounds made according to a unified technique.

On Day 0 and before euthanasia on Days 3, 7, and 14, the microhemocirculation of the anesthetized animals was examined using the MP150 hardware-software complex for electrophysiological studies (BIOPAC Systems, Inc., Goleta, CA, USA), which includes a module for laser-Doppler flowmetry (LDF100C), a sensor for laser-Doppler flowmetry (TSD145), and AcqKnowledgeversion 4.4.1 software. A needle sensor was installed in the center of each side of a standard square wound (1.5–2 mm from its edge) to record frequency shifts in the reflected signal which, in accordance with the software signal processing algorithm, were recorded in perfusion units (Blood Perfusion Units; BPU). The final microperfusion index is the average BPU from all four sides of each wound. On Day 0, the median index of microhemoperfusion on the wound sides was 105–121 BPU (interquartile range Q1: 79–96 BPU; Q3: 139–157 BPU). On Day 0, the average microhemoperfusion index was 115 BPU (Me) with an interquartile range of 96:147 BPU.

The values of the wound sizes and microhemoperfusion indexes on Day 0 in the study groups were statistically indistinguishable.

For histological analysis, three consecutive sections of each wound were removed from euthanized animals on Days 3, 7, and 14. Sections were stained using three different methods: hematoxylin-eosin (for analysis using descriptive light microscopy to assess wound epithelization); hematoxylin (for processing using Image-J software (Version 1.8.0_172, National Institutes of Health, Bethesda, MD, USA) for quantitative analysis of fibroblast and leukocyte cells according to their morp hological characteristics on the granulation layers); and Van Gieson’s stain (to assess the collagenization of the wound, including the location of collagen, the volume, and degree of its maturity). For each section, three areas of equal length were identified (the wound center and two opposite edges). Microscopy was performed at magnifications of 40×, 100×, and 400× using Leica CME (Germany) and Levenhuk D740 (USA) microscopes. The morphologists were blinded concerning which wound sections represented which group.

Statistical analysis of the research results was carried out using SPSS 23.0 software (IBM Corp., Armonk, NY, USA) using standard parametric and nonparametric criteria. The differences were considered significant at *p* < 0.05. The normal distribution of the results was checked using the Kolmogorov-Smirnov test. Descriptive statistics of continuous quantitative data are presented as the mean and standard error of the mean for data with normal distribution, as well as a median (Me), a lower Q1 (25%), and an upper Q3 (75%) quartile for data with non-normal distribution. The Mann-Whitney U test was used to compare two independent nonparametric samples. To compare more than two independent samples, the Kruskal-Wallis test was used. The Wilcoxon test was used to compare two dependent samples. Qualitative indicators were analyzed as a percentage; the comparative analysis of qualitative data was performed using the X^2^ criterion (analysis of contingency tables). To determine the relationship between different indicators, a correlation analysis was carried out using the Pearson and Spearman methods.

## 3. Results

### 3.1. Results of the Wound Size Dynamics Assessment

**Figure 1:**
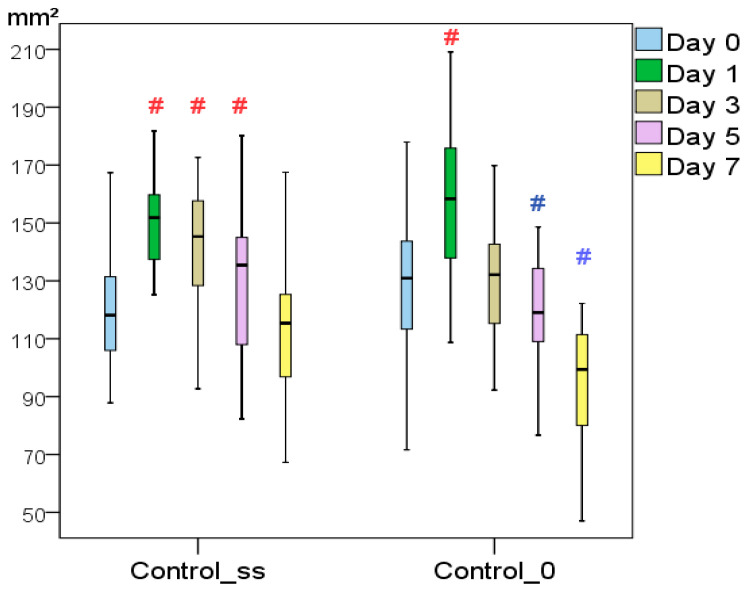
The wound area dynamics of the Control_0 and Control_ss groups throughout the study (# difference in indicators relative to Day 0 within one group at *p* < 0.05; the Wilcoxon test).

Over the course of the study, it was found that intact acute skin wounds (without treatment, C0) demonstrated a 1.31-fold decrease in wound area by Day 7 (*p* < 0.05); over a period of 2 weeks, the wound areas the C0 group decreased 5.94-fold on average (*p* < 0.05). On the last day of the experiment, the wound areas of the C0 group averaged 22.1 mm^2^ in size with an interquartile range of [15.5:30.6] mm^2^. Visual signs of inflammation, including an increase in the wound size, were recorded up to Day 3; positive changes were noticeable from Day 5. In the Css group, a significant increase in the wound area was recorded for 5 days. The area returned to its initial size on the 7th day. On Day 7, the wound areas in the Css group were statistically the same as on Day 0. In the period comprising Days 1–7, the wound areas of the Css group were found to be 22–26% larger than those in the C0 group (*p* < 0.05). Although there were no statistically significant differences between the groups (*p* < 0.05 according to the Mann-Whitney *U* test), the results obtained suggest that even a single additional trauma caused by an injection to the wound corners led to prolonged inflammation and a delay in the onset of regeneration (Figure 1). As a result, by the last day, the Css wound areas averaged 25.9 mm^2^ [18.4:29.0] mm^2^, having decreased by 4.56-fold (in the C0 group, this regression was 1.3 times greater; *p* < 0.05).

The DCh and DHCB treatments were not very effective, with better reduction in the wound area in comparison with only the Control_ss group, and only on the 14th day of the experiment. Over 2 weeks, the wound areas in the DCh group decreased on average 6.71-fold, and in the DHCB group, 7.68-fold, which were respectively 1.46 and 1.68 times better than in the Control_ss group; however, no differences from the Control_0 group were established at any point during the study (*p* < 0.05). The DCh treatment tends to slow wound healing up to 7 days. The wound areas in the DCh group returned to their initial size 2 days later compared to those in the C0 group (on Day 5, the DCh wound area was 1.17-fold larger than the C0 wound area, and on Day 7, it was 1.09-fold larger; *p* < 0.05). In the DHCB group, a significant increase in the wound area was observed after 3 days, with a return to the original size on Day 5; for measurements up to 7 days, the wound size did not differ from the Css group (*p* < 0.05).

The use of MSC demonstrated significant efficacy on Days 1–5 for locally administered MSC and on Days 1–3 for the systemically administered Control_msc group. This provided evidence for the inhibition of pathophysiological inflammatory processes in the acute wound healing phase, which, as a result, improved the process of wound regeneration by the end of the study. The wound areas of the MSC and Control_msc groups, in contrast to those of the C0 and Css groups, did not increase in size during Days 1–3. Their reduction started earlier, from Day 3, i.e., 2 days earlier than in the C0 group and a week earlier than in the Css group. By the 14th day, the MSC wound areas averaged 15.3 mm^2^ [10.5:21.2] mm^2^, having decreased 8.8-fold compared to their initial size. The Control_msc group wound areas averaged 11.3 mm^2^ [9.0:24.3] mm^2^, having decreased 11.9-fold in 2 weeks. Both groups demonstrated a significant improvement (1.9- to 2.0-fold higher) in healing outcome relative to the C0 and Css groups (*p* < 0.05). The statistical insignificance of the wound sizes in the MSC and Control_msc groups at all points of the study indicates similar local and systemic effects of cell therapy.

The effectiveness of the polymer product (PCCD and PCCD + MSC groups) turned out to be most pronounced when used in combination with mesenchymal cells (PCCD + MSC). The wound areas in the PCCD group were significantly smaller compared to the control wounds on Days 1, 3, and 14 (*p* < 0.05) and the wound areas in the PCCD + MSC group were smaller than those in the C0 and Css groups on Days 1, 3, 5, 7, and 14 (*p* < 0.05). On the 14th day, the PCCD wound areas averaged 11.9 mm^2^ [7.7:17.5] mm^2^ and decreased by 11.84-fold over 2 weeks (*p* < 0.01). On the 14th day, the C0 group wound areas were 1.86-fold larger (*p* < 0.01), and those in the Css group were 2.18-fold larger (*p* < 0.01) than in the PCCD group. The wounds of the PCCD + MSC group averaged 8.5 mm^2^ [5.7:11.6] mm^2^ on the 14th day, decreasing 16.73-fold (*p* < 0.01), which represented the maximum effect among all of the groups. In the C0 group, the Day 14 wound area was 2.60-fold larger (*p* < 0.01), and in the Css group, 3.05-fold larger (*p* < 0.01) than in the PCCD + MSC group. Compared with the PCCD + MSC group wound area, the PCCD wound areas were 1.26-fold larger on the 7th day (*p* < 0.05), and 1.40-fold larger on the last day of the study (*p* < 0.05). The greatest difference from the control wounds was achieved on Days 7–14, demonstrating that PCCD + MSC treatment reduced the wound areas by 19.5% on Day 7 and 29.2% on Day 14 compared to the group treated with PCCD only.

**Figure 2:**
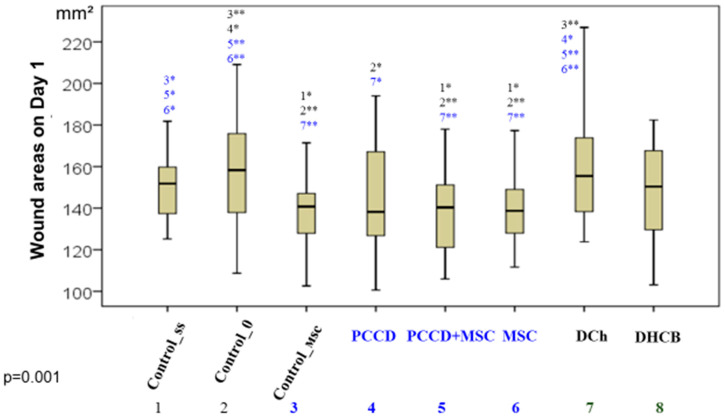
The wound areas in the different groups by the end of the 1st day of the study (* significant difference in the indicator between the Css (1), C0 (2), Control_msc (3), PCCD (4), PCCD + MSC (5), MSC (6), DCh (7), and DHCB (8) groups at *p* < 0.05; ** *p* < 0.01; Kruskal-Wallis test).

Comparative analysis of the wound sizes in all groups at the same time (Kruskal-Wallis test) revealed significant differences beginning on Day 1 (*p* = 0.001), as well as on Day 3 (*p* = 0.0001), Day 5 (*p* = 0.009), Day 7 (*p* = 0.001), and Day 14 (*p* = 0.0001). The best results (smallest wound sizes) on Day 1 were in the PCCD + MSC, MSC, Control_msc, and PCCD groups; the worst results were in the DCh and C0 groups (Figure 2).

**Figure 3:**
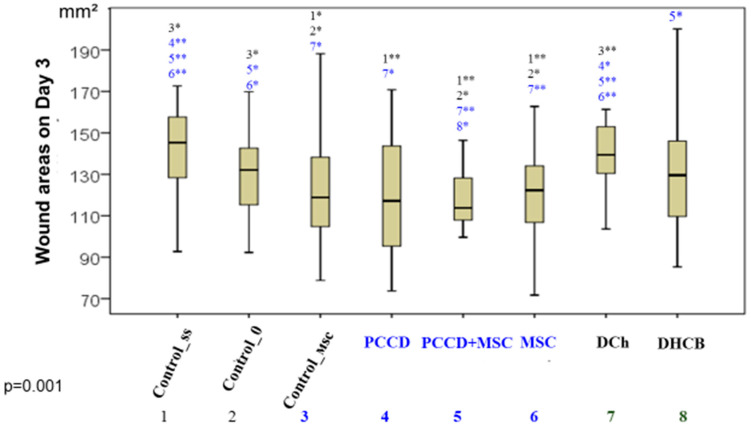
The wound areas in the different groups by the end of the 3rd day (* significant difference in the indicator between the Css (1), C0 (2), Control_msc (3), PCCD (4), PCCD + MSC (5), MSC (6), DCh (7), and DHCB (8) groups at *p* < 0.05; ** *p* < 0.01; Kruskal-Wallis test).

By the end of Day 3, the wounds were distributed in ascending order as follows: DCh = Css < C0 = DHCB < PCCD ≥ MSC ≥ Control_msc < PCCD + MSC (Figure 3).

**Figure 4:**
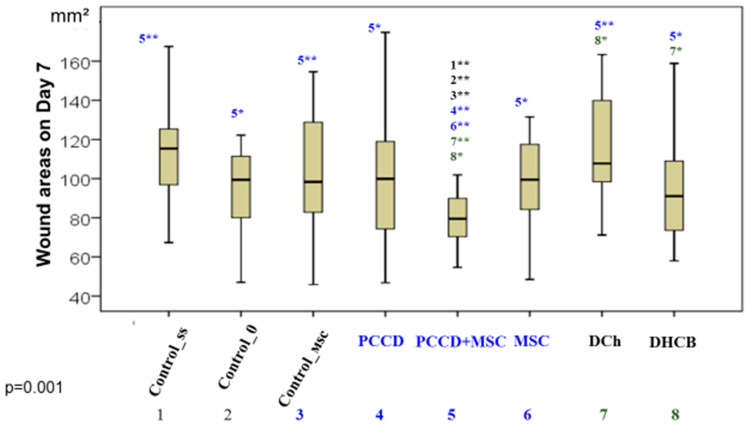
The wound areas in the different groups on Day 7 (* significant difference in the indicator between the Css (1), C0 (2), Control_msc (3), PCCD (4), PCCD + MSC (5), MSC (6), DCh (7), and DHCB (8) groups at *p* < 0.05; ** *p* < 0.01; Kruskal-Wallis test).

On the 7th day of the study, the best group included only the wounds of the PCCD + MSC group, which were significantly different from those of all other groups (Figure 4).

**Figure 5:**
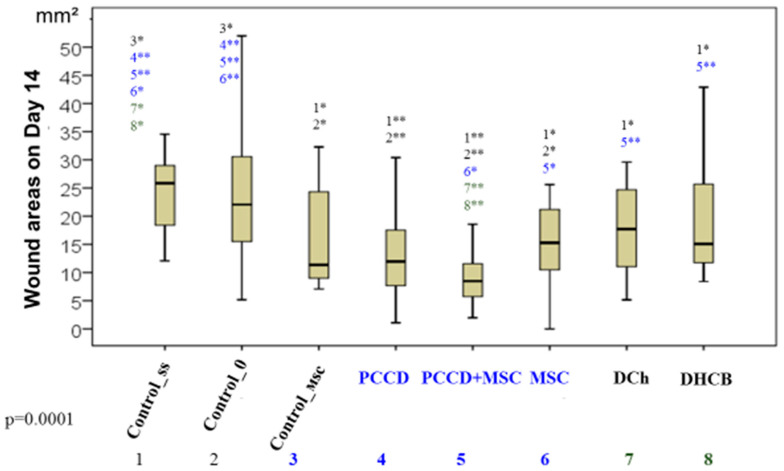
The wound areas in the different groups on Day 14 (* significant difference in the indicator between the Css (1), C0 (2), Control_msc (3), PCCD (4), PCCD + MSC (5), MSC (6), DCh (7), and DHCB (8) groups at *p* < 0.05; ** *p* < 0.01; Kruskal-Wallis test).

On Day 14, the groups were ranked by wound area from worst to best: Css < C0 < DHCB = DCh < PCCD = Control_msc = MSC < PCCD + MSC (Figure 5).

**Figure 6:**
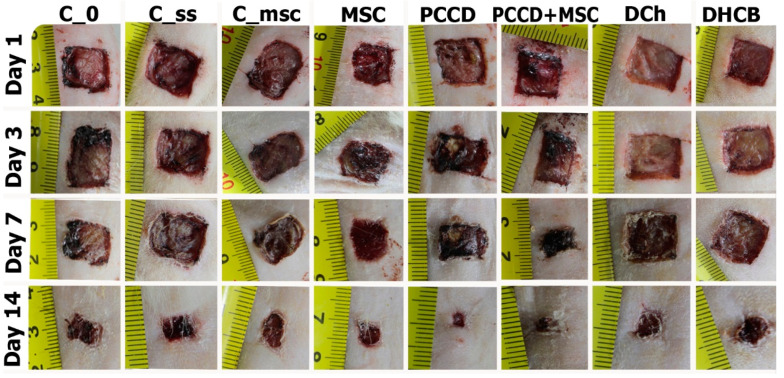
The wound macro images in the different groups during the course of healing.

Thus, among all the wound treatment options we studied, the best result was achieved with the use of PCCD + MSC, from which we conclude that treatment with PCCD + MSC accelerates the healing of skin wounds. Examples of the wound macro images in the different groups during the course of healing are shown in the Figure 6.

### 3.2. Results of the Microhemocirculation Study of the Wound Edge Skin

**Figure 7:**
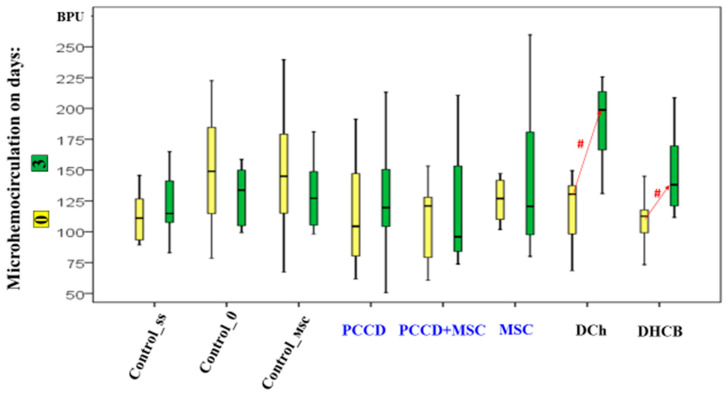
Microhemocirculation dynamics on Day 0 and Day 3; ^#^ difference within a separate group relative to Day 0 at *p* < 0.05 (the Wilcoxon test).

On the 3rd day of the study, the perfusion level had increased in the DHCB and DCh wounds (the microhemocirculation level of the DCh group wound edges was 1.5- to 2.1-fold higher than in all other groups; *p* < 0.05) (Figure 7). The increased hemoperfusion in the wound edges on Day 3 relative to Day 0 correlates with an increase in the wound size and is an early marker of an unfavorable prognosis.

**Figure 8:**
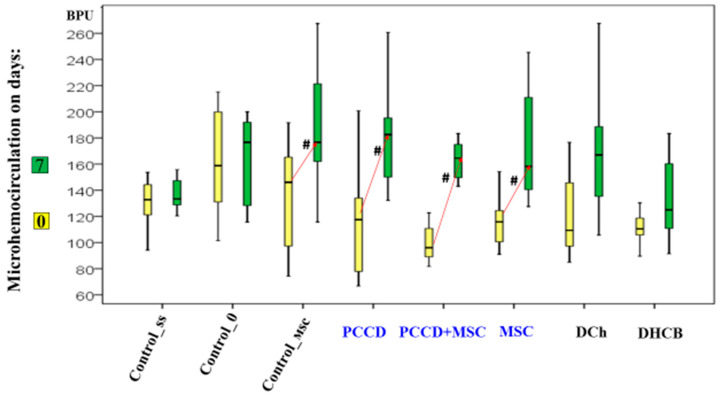
Microhemocirculation dynamics on Days 0 and 7; ^#^ difference within a separate group relative to Day 0 at *p* < 0.05 (the Wilcoxon test).

On Day 7, the hemoperfusion level did not change relative to Day 0 in the control wound areas. It increased in the MSC and Control_msc wound areas by 1.2- to 1.4-fold (*p* < 0.05) and most of all in the PCCD + MSC and PCCD wound areas (1.6- to 1.7-fold; *p* < 0.05). In the DHCB and DCh wound areas, the recorded positive perfusion effect ended and became equal to that in control animals by the 7th day, thus, no differences were observed compared to Day 0 (Figure 8).

**Figure 9:**
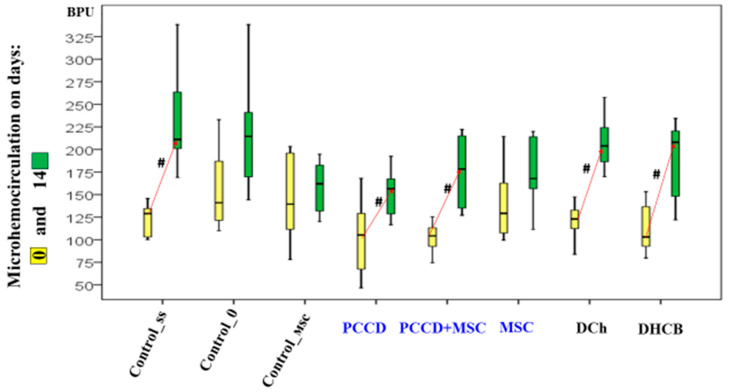
Microhemocirculation dynamics on Days 0 and 14; ^#^ difference within a separate group relative to Day 0 at *p* < 0.05 (the Wilcoxon test).

On the 14th day, the microhemocirculation index increased relative to the modeling day; the most significant increases (1.5- to 2.0-fold on average) were recorded in the Css, DCh, DHCB, PCCD, and PCCD + MSC groups. The maximum perfusion level in the wound areas was observed in the C0 and Css groups, exceeding on average 1.2–1.4 times that in the Control_msc, MSC, PCCD + MSC, and PCCD groups (this indicator was the lowest in these groups); this level was not statistically different from that in the DCh and DHCB groups (Figure 9).

The correlation analysis showed significant direct relationships of moderate strength between the microhemocirculation level and the wound area on Day 14 (r = 0.403; *p* < 0.01). In other words, a low level of tissue perfusion at the wound edge was associated with an accelerated regeneration rate, a marker of a favorable prognosis confirmed by morphometric study of histopreparations.

### 3.3. Results of the Histological and Morphological Assessments

**Figure 10:**
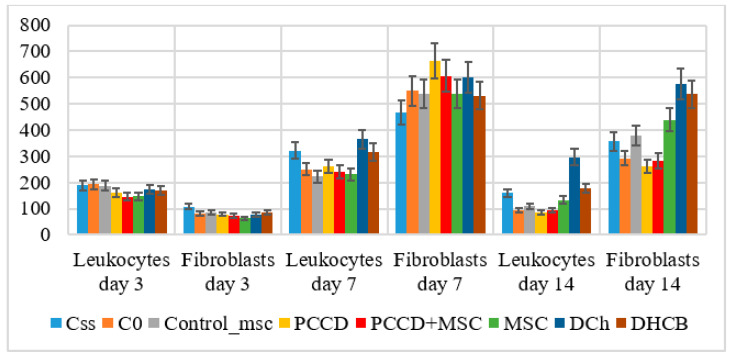
The number of leukocytes and fibroblasts in the wound tissue sections on Days 3, 7, and 14 (on average for all wound sections, abs.).

The histological examination on Day 3 demonstrated several signs of exudative inflammation, marked by an expansion and plethora of blood vessels, tissue edema, emigration of leukocytes from the bloodstream, and granulation tissue infiltration. In different wound sections, the number of leukocytes was 1.8- to 2.3-fold higher than that of fibroblasts (*p* < 0.05), varying by group. The most significant wound healing mechanisms took place in the central wound tissues, where the number of leukocytes was 1.2- to 1.9-fold higher than the number found at the wound periphery (*p* < 0.05). The smallest number of leukocytes recorded on Day 3 was in the MSC, PCCD + MSC, and PCCD groups. In the wound center, smaller wound size correlated with a lower concentration of leukocytes and a higher percentage of fibroblasts. Moreover, it was associated with a faster transition from an exudative to a proliferative phase, mostly observable in the PCCD + MSC group. On Day 7, the proliferative phase was determined histologically in the wound tissues, where it was observed that new tissues had formed, abundantly infiltrated with cells of different types and degrees of maturity. The total number of cells increased relative to Day 3 in the different groups by 2.0- to 3.9-fold in the center (*p* < 0.05) and by 3.0- to 6.5-fold at the wound edges (*p* < 0.05). Thus, on the 7th day, the processes occurring at the wound edges played the greatest role in wound healing. An increase in the number of leukocyte cells (on average, 1.18- to 2.09-fold in different groups), and especially in the number of fibroblast cells (on average, 4.3- to 8.6-fold in the different groups; *p* < 0.05) was observed. Therefore, on Day 7, the healing was carried out mainly by fibroblasts producing extracellular matrix, including collagen. The number of fibroblasts on the 7th day in different wound sections was 1.5- to 2.5-fold higher than that of leukocytes (*p* < 0.05) (Figure 10).

**Figure 11:**
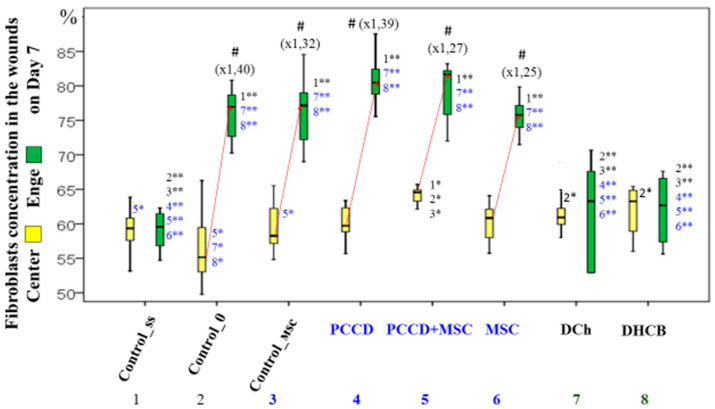
The proportion of fibroblasts in the central (yellow) and wound edges (green) tissues of different groups (%). Significance of intergroup differences according to post hoc tests: * *p* < 0.05; ** *p* < 0.01. ^#^ significant difference in the fibroblast proportion of the central and edge tissues within the same group at *p* < 0.05.

By the end of Day 7, the total cell number had increased the least in the Css group (on average, 2.04-fold in the center and 3.01-fold at the edges), and the most in the PCCD group (on average, 3.92-fold in the center and 6.49-fold at the edges; *p* < 0.05) and PCCD + MSC group (3.32-fold in the center and 5.55-fold at the edges; *p* < 0.05). A consistently high level of leukocyte infiltration on Day 7 served as a marker of persistent inflammation and a slowing of the repair process. On the other hand, a low concentration of leukocytes at the wound edges provided a useful marker of accelerated healing. In the case of naturally well-healed wounds, the number of leukocytes at the edges of the wound areas was half that observed in the wound center, with the main role in the regeneration process played by the fibroblasts. The fibroblast numbers were the highest in the PCCD and PCCD + MSC groups, and a significantly important predominance of fibroblasts compared to other cells in the wound edges, rather than in the center, was found in the C0, MSC, Control_msc, PCCD, and PCCD + MSC groups (Figure 11).

Wounds with the highest concentration of fibroblasts and the lowest numbers of leukocytes had regenerated better by Day 7, especially at the wound edges. This was confirmed by the results of the correlation analysis of wound size indicators and morphometric data examined on the 7th day of the experiment (Table 1).

The use of PCCD and PCCD + MSC activated proliferative processes and increased the number of fibroblasts, their accelerated maturation, and the production of extracellular matrix proteins. In terms of morphometry, the MSC and Control_msc groups did not differ from the C0 group on Day 7. The positive healing results in these groups on the 7th day are associated rather with the effect of leukocytes during an earlier healing phase. Leukocytes migrate faster into the wound, providing the MSC anti-inflammatory effect and, simultaneously, accelerated transition to the proliferative phase, without directly affecting the fibroblasts. We also observed no significant effect on the presence of fibroblasts on Day 7 in the DCh and DHCB groups. In the DCh group wounds, the percentage of fibroblasts was low, while the percentage of leukocytes was high. In addition, there was no increase in the number of fibroblasts in the wound center, which was also typical for the C0 group, indicating the ineffectiveness of this drug. The introduction of DHCB via injection to the wound edges increased the inflammatory response and delayed the regeneration processes relative to those in the non-invasive groups, so the morphometric parameters did not differ from those of the Css group (*p* < 0.05).

**Figure 12:**
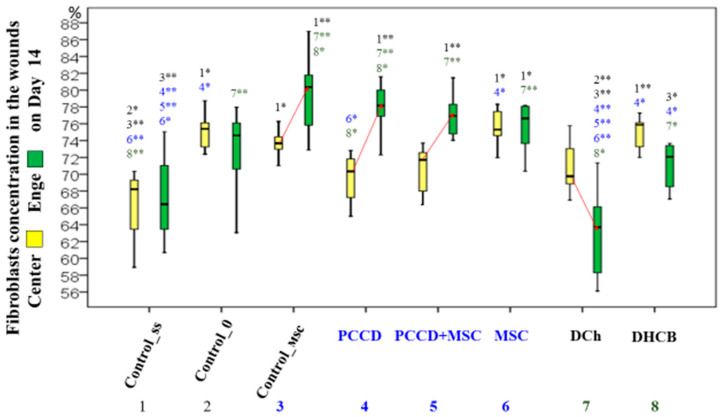
The proportion of fibroblasts in the central (yellow) and wound edges (green) tissues of different groups (in %). Significance of intergroup differences: * *p* < 0.05; ** *p* < 0.01. Arrows indicate a significant difference in the proportion between the center and the edges within one group at *p* < 0.05.

On Day 14, the total number of all cells decreased significantly, 1.5-fold on average, compared to Day 7 (1.1- to 3.1-fold in the center and 1.1- to 3.2-fold in the edges). The number of leukocytes decreased in particular, which correlated directly with the reduction in the wound area size. The ratio of central-to-edge cells leveled off, indicating the same intensity of regenerative processes in all parts of the wound. The greatest decrease was observed in the center and edge cell populations in the groups treated with polymeric agents (PCCD, 2.98-fold; PCCD + MSC, 2.04-fold; *p* < 0.05). The smallest change was seen in the DCh and DHCB groups. The highest concentration of leukocytes was recorded in the DCh group; the concentration of fibroblasts on the 14th day was 2.9 times higher than that of the leukocytes, reaching the maximum as the latter were eliminated. The concentration of fibroblasts was higher in smaller wounds. In the MSC and DHCB injection groups, the number of fibroblasts in the center and edge tissues was higher than in the Css group, which was reflected in the general and morphological records. The non-invasive groups did not differ from C0 for this parameter. The exception was for wounds in the DCh group, the edges of which contained a significantly lower percentage of fibroblasts than those in other groups (Figure 12).

During the correlation analysis it was found that by Day 14, the wounds healed better if they contained fewer cells in all wound sections, and the percentage of fibroblasts was greater than the concentration of leukocytes (Table 2).

**Figure 13:**
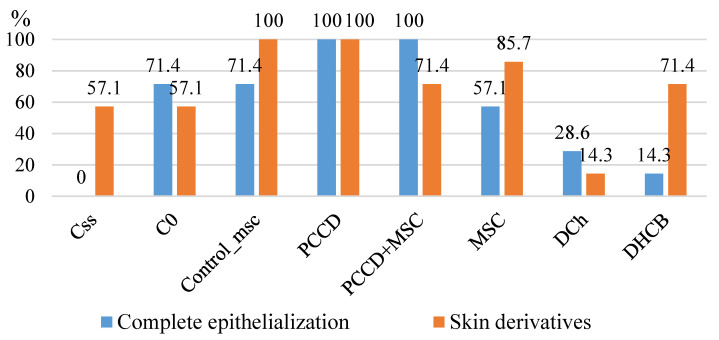
Frequency graphic showing complete wound surface epithelialization and detection of 14th day of the study.

The morphometric results correlated with better healing outcomes. By Day 14 in the PCCD + MSC and PCCD groups, 100% of the wounds were epithelialized (all sections covered with stratified keratinizing epithelium), and we observed the largest number of formed skin derivatives (86–100%) as well as the best formation and maturation of the collagen matrix (Figure 13).

In the course of the morphometry and microcirculation correlation analysis performed on the wound samples from euthanized animals on the 3rd, 7th, and 14th days, many reliable relationships were established. The high level of hemoperfusion around the wounds on Day 3 directly correlated with the leukocyte numbers in the wound edges (r = 0.289; *p* < 0.05), in the wound as a whole (r = 0.254; *p* < 0.05), and with the average fibroblast/leukocyte ratio for the animals withdrawn on Day 3 (r = −0.215; *p* < 0.05).

Thus, rising leukocyte infiltration correlated with the growing volume of wound hemoperfusion on the 3rd day. On the 7th day of the study, it was found that high hemoperfusion levels also directly correlated with the number of fibroblasts in the wound edges (r = 0.239; *p* < 0.05), and with their percentage in the wound edge fragments (r = 0.302; *p* < 0.05), in the wound segments on average (r = 0.274; *p* < 0.05), with the fibroblast/leukocyte ratio in the wound edges (r = 0.292; *p* < 0.05), and in the wound as a whole (r = 0.262; *p* < 0.05). In contrast with Day 3, the increasing microhemoperfusion levels observed on Day 7 were associated with an increase in the concentration of fibroblasts, which predetermined the acceleration of wound healing. On Day 14, high levels of hemoperfusion around the wound skin directly correlated with the number of leukocytes in all wound sections (in the center, edges, and the whole wound: r = 0.258–0.318; *p* < 0.05) and with the percentage of leukocytes in all sections (r = 0.346–0.389; *p* < 0.05). Furthermore, there was an inverse correlation between hemoperfusion and the fibroblast/leukocyte ratio in the wound edges (r = −0.322; *p* < 0.05). We also observed that prolonged inflammatory reactions, characterized by an excessive number of leukocytes and lasting up to 14 days, correlated with a high level of microhemoperfusion index around the wound. The strongest correlation was recorded for the wound edges on the 14th day.

## 4. Discussion

When healing without treatment under a scab, an acute wound tends to demonstrate visual signs of inflammation for up to 3 days and positive changes from the 5th day. In our untreated wounds, we observed that the wound size had decreased 1.31-fold after 7 days, and 5.94-fold after 2 weeks. Wound trauma caused by even a single additional injection prolonged inflammation and delayed the onset of the regeneration period by 5–7 days, reducing the efficiency of healing 1.3-fold by Day 14 (a reduction of 4.56-fold after 2 weeks).

The use of deproteinized hemoderivative of calf blood did not demonstrate any effect on the wound size dynamics until the end of the exudative phase and the transition to the proliferative phase of inflammation. The use of Levomekol^®^ was accompanied by an increase in the size of the acute wounds for up to 3 days and a delay in regeneration for at least 2 days compared with results in the control groups.

The use of mesenchymal stem cells demonstrated its effectiveness on Days 1–5 for local and Days 1–3 for systemic use, improving the regeneration process 1.9- to 2.0-fold by the end of the experiment. The wound healing dynamics were comparable between the local and systemic application of MSC.

The use of PCCD for 2 weeks resulted in an 11.8-fold reduction in wound size, which is twice that of the control group. The maximum efficiency was observed in wounds treated with PCCD + MSC (16.7-fold wound reduction after 14 days; 2.6 times higher than in the control group), with accelerated healing processes in both the exudative and proliferative phases.

Increased microhemoperfusion at the wound edges after 3 days is a sign of ineffective treatment; coupled with continued active exudative inflammation, this correlated with an increase in the wound size. Increased microhemoperfusion after 7 days is a sign of that the transition from the exudative to the proliferative phase has been completed, resulting in accelerated wound healing. A high level of hemoperfusion on the 14th day correlated directly with the largest wound sizes and was associated with inhibited regeneration.

After 3 days, the number of leukocytes exceeded the number of fibroblasts by 1.8- to 2.3-fold; in the wound center, there are 1.2- to 1.9-fold more leukocytes than on its edges. The smallest wound size correlated with the lowest leukocyte concentration and the highest percentage of fibroblasts in the central tissues of the wounds. Specifically, low levels of leukocyte infiltration in the MSC, PCCD + MSC, and PCCD groups were associated with an anti-inflammatory effect and an acceleration of the transition to the cell proliferative phase. The fastest transition was observed in the PCCD + MSC group.

After 7 days, wound healing depends mainly on wound edge regeneration mechanisms, including participation by the fibroblasts, which were 1.5- to 2.5-fold more abundant than leukocytes; the concentration of fibroblasts at the wound edges was 1.2- to 1.4-fold higher than in the center. The best healing effect was seen in the wounds containing the highest number of fibroblasts (especially at the edges) and the lowest number of leukocytes. The highest number of fibroblasts of different degrees of maturity was recorded on the 7th day in the PCCD + MSC and PCCD groups. A consistently high level of leukocyte infiltration on Day 7 serves as a marker of persistent inflammation and slowed repair. A low concentration of leukocytes, especially at the wound edges, is a marker of accelerated healing.

On the 14th day, the number of cells in the wound tissues of all groups was reduced 1.5-fold compared to Day 7, particularly the number of leukocytes, which correlated directly with the reduction in the wound area. The regenerative processes were comparable among all wound sections. The concentration of fibroblasts on the 14th day was 2.9-fold higher than that of the leukocytes, reaching the maximum percentage due to the elimination of the latter. The cell concentration decreased the most in the PCCD and PCCD + MSC groups, and the least in the DCh group (in this group, the concentration of leukocytes was the highest). The morphometric result correlated with the best results in the PCCD and PCCD + MSC groups (by Day 14, 100% of the wounds in both groups were covered with stratified keratinized epithelium), which had the largest number of formed skin derivatives (86–100%) and demonstrated density and maturity in the newly synthesized collagen.

## 5. Conclusions

Our findings allow us to make the following practical recommendations.

Injections of medical substances into the wound edges can slow the regeneration process; therefore, the number of such manipulations should be limited as much as possible.

Antimicrobial drugs (particularly Levomekol^®^) should not be used widely in the treatment of acute wounds without signs of bacterial contamination, as they slow the healing process. DHCB injections for the treatment of acute wounds without ischemia should also be avoided since they do not affect the wound healing rate during the first week.

Because the maximum efficiency of mesenchymal stem cells is limited to the first 3 days for systemic use and the first 5 days for a local application, it is advisable to inject them 5–7 days later to increase the specific effect of MSC.

Proven preclinical efficacy of the previously developed polymeric compounds based on chitosan and cellulose enriched with cerium dioxide nanoparticles, especially in combination with mesenchymal stem cells, demonstrates the feasibility of further studies on the pathogenetic mechanisms and safety of these compounds for subsequent use in clinical practice.

A non-invasive assessment of the microhemocirculation level of the wound edges allows a prognosis to be reached concerning the results of wound healing. This method can be recommended for the optimal selection of a drug or combination of drugs, for monitoring the efficacy of treatment, and for improved acceleration of the regeneration process.

To increase the effectiveness of treatments for acute wounds, when choosing a medicine, one should assess not only the wound condition and the phase of the healing process, but also the pharmacological mechanisms and biological properties of the agent itself.

## Figures and Tables

**Table 1:** The correlation analysis of the wound size and morphometric indicators on Day 7 (values of r < 0.2 are presented at *p* < 0.05).

Wound Section	Indicator	Wound Area Day 7 (mm^2^)	Area Change on Day 7 (S0c—S7c; mm^2^)
Center	Cells total (abs.)	---	−0.346 **
	Fibroblasts (abs.)	---	−0.354 **
	Leukocytes (abs.)	---	−0.259 **
Edge	Fibroblasts (abs.)	−0.213 *	−0.504 **
	Leukocytes (abs.)	0.354 **	0.564 **
	% Fibroblasts	−0.360 **	−0.607 **
	% Leukocytes	0.360 **	0.607 **
	Fibroblasts/Leukocytes	−0.318 *	−0.578 **
On average within a wound	Fibroblasts (abs.)	−0.282 *	−0.478 **
	Leukocytes (abs.)	0.255 *	0.401 **
	% Fibroblasts	−0.363 **	−0.598 **
	% Leukocytes	0.363 **	0.598 **
	Fibroblasts/Leukocytes	−0.331 **	−0.554 **

Note: * *p* < 0.05; ** *p* < 0.01.

**Table 2:** The correlation analysis of the wound size and morphometric indicators on Day 14 (values of r < 0.2 are presented at *p* < 0.05).

Wound Section	Indicator	Wound Area Day 14 (mm^2^)	Area Change on Day 14 (S0c—S7c; mm^2^)
Center	Cells total (abs.)	0.304 *	0.305 *
	Fibroblasts (abs.)	0.273 *	0.262 *
	Leukocytes (abs.)	0.356 **	0.389 **
Edge	Cells total (abs.)	0.331 *	0.341 *
	Fibroblasts (abs.)		0.242 *
	Leukocytes (abs.)	0.424 **	0.439 **
	% Fibroblasts	−0.480 **	−0.437 **
	% Leukocytes	0.480 **	0.437 **
	Fibroblasts/Leukocytes	−0.434 **	−0.345 **
On average within a wound	Fibroblasts (abs.)	0.288 *	0.275 *
	Leukocytes (abs.)	0.385 **	0.420 **
	% Fibroblasts	−0.372 **	−0.407 **
	% Leukocytes	0.368 **	0.405 **
	Fibroblasts/Leukocytes	−0.353 **	−0.363 **

Note: * *p* < 0.05; ** *p* < 0.01.

## Data Availability

Details regarding and data supporting reported results can be found from the first authors.
